# PI3K-AKT, JAK2-STAT3 pathways and cell–cell contact regulate maspin subcellular localization

**DOI:** 10.1186/s12964-021-00758-3

**Published:** 2021-08-14

**Authors:** M. T. Longhi, L. E. Silva, M. Pereira, M. Magalhães, J. Reina, F. N. L. Vitorino, B. M. Gumbiner, J. P. C. da Cunha, N. Cella

**Affiliations:** 1 Instituto de Ciencias Biomedicas, Departamento de Biologia Celular e do Desenvolvimento, Universidade de Sao Paulo, Av. Prof. Lineu Prestes, 1524, São Paulo, SP 05508-000 Brazil; 2 Department of Pediatrics and Biochemistry, University of Washington School of Medicine, Center for Developmental Biology and Regenerative Medicine, Seattle Children’s Research Institute, 1900 9th Ave. Mailstop JMB-5, Seattle, WA 98101 USA; 3 Laboratório de Ciclo Celular, Center of Toxins, Immune Response and Cell Signaling-CeTICS, Instituto Butantan, Av. Vital Brasil, 1500, São Paulo, SP 05503-900 Brazil

**Keywords:** Maspin, EGFR, PI3K-AkT, JAK2-STAT3, Cell-to-cell contact

## Abstract

**Background:**

Maspin (SERPINB5) is a potential tumor suppressor gene with pleiotropic biological activities, including regulation of cell proliferation, death, adhesion, migration and gene expression. Several studies indicate that nuclear localization is essential for maspin tumor suppression activity. We have previously shown that the EGFR activation leads to maspin nuclear localization in MCF-10A cells. The present study investigated which EGFR downstream signaling molecules are involved in maspin nuclear localization and explored a possible role of cell–cell contact in this process.

**Methods:**

MCF-10A cells were treated with pharmacological inhibitors against EGFR downstream pathways followed by EGF treatment. Maspin subcellular localization was determined by immunofluorescence. Proteomic and interactome analyses were conducted to identify maspin-binding proteins in EGF-treated cells only. To investigate the role of cell–cell contact these cells were either treated with chelating agents or plated on different cell densities. Maspin and E-cadherin subcellular localization was determined by immunofluorescence.

**Results:**

We found that PI3K-Akt and JAK2-STAT3, but not MAP kinase pathway, regulate EGF-induced maspin nuclear accumulation in MCF-10A cells. We observed that maspin is predominantly nuclear in sparse cell culture, but it is redistributed to the cytoplasm in confluent cells even in the presence of EGF. Proteomic and interactome results suggest a role of maspin on post-transcriptional and translation regulation, protein folding and cell–cell adhesion.

**Conclusions:**

Maspin nuclear accumulation is determined by an interplay between EGFR (via PI3K-Akt and JAK2-STAT3 pathways) and cell–cell contact.
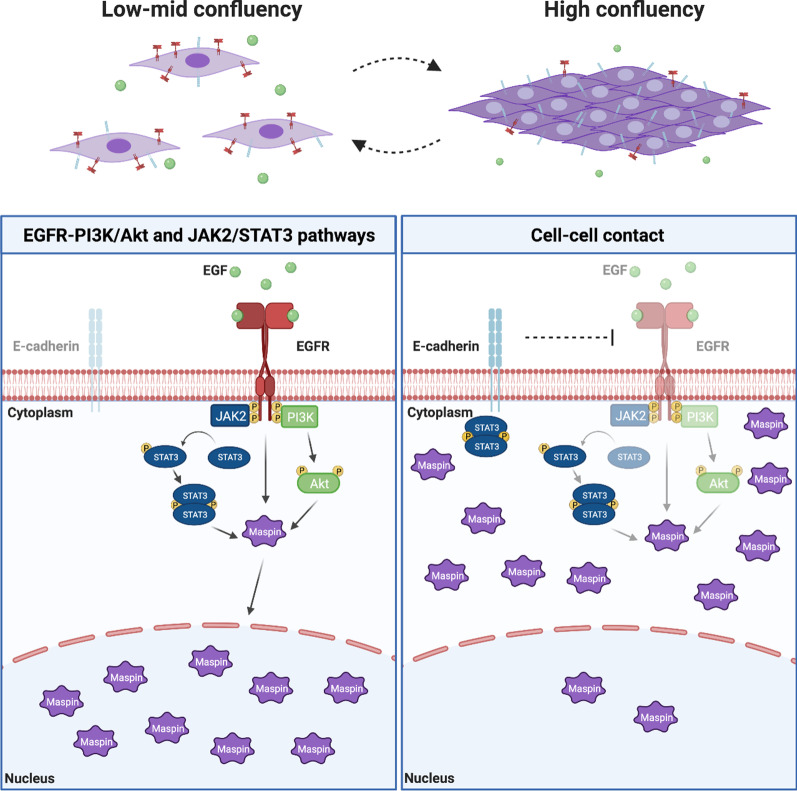

**Video Abstract**

**Supplementary Information:**

The online version contains supplementary material available at 10.1186/s12964-021-00758-3.

## Background

Maspin (SerpinB5) is a 42 kDa serpin (serine protease inhibitor) which does not inhibit proteases [1]. Maspin regulates cell adhesion, migration, invasion, proliferation and cell death [2–5], which are consistent with its role as a tumor growth and metastasis suppressor [4]. Clinical studies, however, brought controversies to the field, as maspin was found to be associated with aggressive tumors and poor prognosis [6–9]. Another set of studies looked at maspin subcellular localization rather than expression. It has been reported that maspin nuclear localization is essential for its tumor suppression activity [10]. Accordingly, clinical studies observed that maspin localization in the nucleus correlates with favorable prognosis in different cancer types [8, 11, 12], whereas cytoplasmic-only or cytoplasmic and nuclear localization correlate with poor prognosis [13–15]. How maspin nuclear localization is regulated and how this localization relates to its tumor suppressor activity are important unresolved questions.

In MCF-10A cells, a non-transformed mammary epithelial cell line which endogenously expresses maspin, epidermal growth factor receptor (EGFR) activation leads to maspin phosphorylation and nuclear accumulation [16]. The objective of this study was to determine which EGFR downstream signaling pathways are responsible for maspin nuclear localization. To shed light on maspin function in the nucleus, we identified a set of maspin-ligands in EGF-treated cells, revealing a role of maspin on previously unrecognized processes like control of gene expression at the RNA level and protein folding. In addition, we provide evidence that maspin nuclear localization is regulated by a crosstalk between EGFR signaling and cell–cell contact.

## Materials and methods

### Cell culture, reagents and treatments

MCF-10A human mammary epithelial cells (from Banco de Células do Rio de Janeiro) were maintained in Dulbecco’s modified Eagle medium (DMEM)/F12 (Invitrogen) containing 5% donor horse serum, 20 ng/mL epidermal growth factor (EGF), 100 ng/mL cholera toxin, 10 µg/mL insulin, 500 µg/mL hydrocortisone, 50 U/mL penicillin, and 50 µg/mL streptomycin. The HaCaT human keratinocyte cell line was kindly provided by Dr Fabio Luis Forti (Chemistry Institute, São Paulo University). These cells were maintained in DMEM containing 10% of fetal bovine serum. Cells were kept at 37 °C in 5% CO_2_ atmosphere. Growth factors and hormones were purchased from Sigma. Table 1 displays the antibodies and pharmacological inhibitors used in this study.

**Table 1:** Antibodies and inhibitors employed in this study

Brand	Antibody/inhibitor	Experiment	Dilution/dose
Millipore (MABC603)	Mouse anti-maspin	WB	1:1000
Sigma (HPA019132)	Rabbit anti-maspin	IF	1:100
Sigma (HPA019125)	Rabbit anti-maspin	IF	1:100
Santa Cruz Biotech (373745)	Mouse anti-EGFR(A-10)	WB; IP	1:1000; 1:50
Cell Signaling (#14472)	Mouse anti-E-cadherin	IF	1:100
Santa Cruz Biotech (5286)	Mouse α-tubulin (clone b7)	WB	1:1000
Santa Cruz Biotech. (47778)	Mouse anti-β-actin	WB	1:1000
Thermo Scientific (mab636)	Mouse anti-lamin A/C	WB	1:1000
Cell Signaling (9275S)	Rabbit anti-p-Akt (T308)	WB	1:1000
Santa Cruz Biotech. (81434)	Mouse anti-Akt1/2/3	WB	1:1000
Sigma	anti-rabbit HRP	WB	1:10,000
Sigma	anti-mouse HRP	WB	1:10,000
Invitrogen	anti-rabbit Alexa Fluor 488	IF	1:500
Invitrogen	anti-mouse Alexa Fluor 594	IF	1:500
AdooQ Biosciences (#A10422)	Gefitinib (Iressa)	EGFR	10 µM
AdooQ Biosciences (#A11161)	Wortmannin	PI3K	10 µM
AdooQ Biosciences (#A11795)	WP1066	JAK2/STAT3	10 µM
AdooQ Biosciences (#A12723)	PP2	Src	10 µM
AdooQ Biosciences (#A12368)	FTI-277	H/K-Ras	10 µM
AdooQ Biosciences (#A13790)	Go6983	PKC	10 µM
AdooQ Biosciences (#A11148)	BX-795	PDK1	5 µM
AdooQ Biosciences (#A10782)	Rapamycin (Sirolimus)	mTOR	10 µM
AdooQ Biosciences (#A15279)	VO-OHpic	PTEN	10 µM
AdooQ Biosciences (#A13210)	Triciribine	Akt	10 µM

*WB* Western blot, *IF* immunofluorescence, *IP* immunoprecipitation

*All inhibitors were dissolved in DMSO

**Fig. 1:**
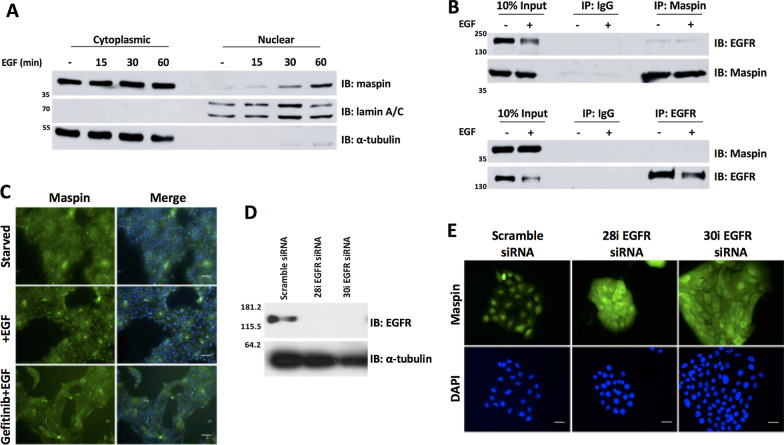
Maspin nuclear translocation depends on EGFR. **A** Starved MCF-10A cells were treated with EGF (20 ng/mL) for the indicated periods of time. Nuclear and cytoplasmic fractions were prepared and maspin protein levels were analyzed by immunoblot. Fractionation efficiency was monitored by reprobing the membrane with anti-lamin A/C and anti-α-tubulin. **B** Starved MCF-10A cells were left untreated (−) or treated with 20 ng/ml EGF for 1 h (+). Whole cell lysates were immunoprecipitated (IP) with anti-maspin or anti-EGFR, as indicated. Isotype matched IgG was used as a negative control. EGFR and maspin co-immunoprecipitation and input samples were evaluated by immunoblot (IB), as indicated on the right side of the figure. **C **Starved MCF-10A cells were pretreated with EGFR inhibitor gefitinib or vehicle (DMSO) for 30 min followed by 20 ng/ml EGF for additional 1 h. Maspin localization was analyzed by immunofluorescence with anti-maspin antibody. **D** MCF-10A cells were transiently transfected with Stealth Select RNAi negative control or two different siRNA against EGFR. EGFR silencing was evaluated by immunoblot with anti-EGFR. α-Tubulin was probed as a loading control. **E** 48 h after transfection cells were treated with 20 ng/ml EGF for 1 h and maspin localization was analyzed by immunofluorescence. Nuclei were stained with DAPI. MW markers are indicated on the left side of the images. Images are representative of at least three independent assays. Scale bar: 20 μm

MCF-10A cells were pretreated with different inhibitors for 30 min followed by 20 ng/ml EGF treatment for additional 1 h. Cells were processed for immunofluorescence analysis as described below. For subcellular fractionation and maspin-EGFR co-immunoprecipitation (Fig. 1A, B), MCF-10A cells were starved from serum and growth factors for 18–24 h. Cells were left untreated or treated with 20 ng/ml EGF during the intervals mentioned in the figures. Protein extraction and further analyses are described below. For EGFR silencing, 5 × 10^4^ cells were plated on a 6-well plate and transfected with 30 pmol of customized siRNA against EGFR (WD02588728 and WD025887300, which target two distinct regions of the EGFR mRNA), or Stealth Select RNAi negative control cat#1299003 (Invitrogen/Thermofisher). Cells were transfected with Lipofectamine® RNAiMax reagent, according to manufacturer’s instructions. After 48 h, cells were starved from both serum and growth factors for 18–24 h and stimulated for 1 h with 20 ng/mL EGF prior to be fixed and processed for immunofluorescence or Western blot analyses. To investigate if EGF regulates maspin subcellular localization in HaCaT cells, cells were serum-starved for 24 h. Cells were then treated with 20 ng/ml EGF for the intervals mentioned in the figure. Finally, cells were fixed and processed for immunofluorescence.

In order to investigate the role of calcium-dependent cell–cell contact, MCF-10A cells were plated in complete medium overnight, and cell–cell junctions were disrupted by incubation with 4 mM EGTA for 30 min at 37 °C [17]. In a different experimental approach, the role of cell–cell contact was investigated by plating MCF-10A and HaCaT cells in complete medium overnight at the following cell densities—1 × 10^4^, 4.5 × 10^4^ and 10 × 10^4^ cells/cm^2^. Cells were fixed and processed for immunofluorescence, as described below.

### Protein extraction, subcellular fractionation and Western blot

For protein extraction, cells were washed twice with ice-cold PBS and lysed in RIPA buffer (50 mM Tris pH 7.4, 1% Triton X-100, 0.1% SDS, 0.5% sodium deoxycholate, 150 mM NaCl, 1 mM EDTA pH 8.0 and 1 mM EGTA) containing protease (1 μg/ml leupeptin, 1 μg/ml aprotinin and 1 mM PMSF) and phosphatase inhibitors (2 mM Na_3_VO_4_, 5 mM NaF and 50 μM β-glycerophosphate). Lysates were centrifuged at 12.000 rpm for 10 min at 4 °C and supernatants were collected and stored at −20 °C. Nuclear and cytoplasmic fractions were obtained according to the protocol adapted from [18], as follows: cells were washed twice with PBS, trypsinized and centrifuged at 600×*g* for 5 min at 4 °C. Supernatants were discarded and cell pellets resuspended in hypotonic buffer A (10 mM Hepes pH 7.9, 10 mM KCl, 0.1 mM EDTA, 0.1 mM EGTA and 1 μM DTT) containing protease and phosphatase inhibitors mentioned above, and incubated on ice for 25 min. Nonidet-P40 at 0.85% final concentration was added to lysate, samples were vortexed for 15 s and centrifuged at 800×*g* for 5 min at 4 °C. Supernatants were collected as cytoplasmic fractions and cell pellets washed twice in buffer A followed by centrifugation. Nuclear pellets were disrupted in ice-cold hypertonic buffer B (20 mM Hepes pH 7.9, 0.4 M NaCl, 1 mM EDTA, 1 mM EGTA, 1 µM DTT) containing the same protease and phosphatase inhibitors described above for 15 min with intermittent vortexing. Finally, the suspensions were centrifuged at 15,000×*g* for 5 min at 4 °C and supernatants were collected as nuclear fractions. Protein quantification was performed using Bradford assay according with manufacture’s protocol. Western blot analysis and quantification was performed as previously described [16].

### Co-immunoprecipitation

MCF-10A cells were treated as described in the first section. Total cell lysates (600 µg) obtained in RIPA buffer were incubated with either 2 µg of anti-maspin antibody (Millipore) or anti-EGFR (Santa Cruz, A-10) and incubated overnight at 4 °C. Protein G Sepharose beads (GE Healthcare, Piscataway, NJ, EUA) were added to samples and incubated for 90 min at 4 °C under gentle agitation. The immunocomplexes were pelleted, beads were washed three times with RIPA buffer and denatured in Laemmli sample buffer for subsequent Western blot analyses [16]. Isotype-matched IgG antibody was used as a negative control.

### Immunofluorescence, image acquisition and quantification

For immunofluorescence experiments, cells were plated on coverslips, washed twice with PBS and fixed in 2% paraformaldehyde (PFA)/PBS for 20 min at room temperature. Cells were permeabilized in 0.5% Triton X-100/PBS for 10 min on ice and blocked with PBS 10% of goat serum for 1 h at room temperature. Primary antibodies were diluted in blocking solution overnight at 4 °C. Alexa Fluor 594- or 488-conjugated secondary antibodies were incubated for 1 h at room temperature. Nuclei were stained with DAPI (1:5000) for 5 min at room temperature and coverslips were mounted in Prolong Gold Antifade mountant (ThermoFisher, MA, USA). Conventional immunofluorescence was performed in a widefield DMi8 Leica fluorescence microscope and images analyzed in Las X software (Leica Microsystems, Germany). All image acquisitions were performed at 63×, 40× or 20× magnification and under the same exposure, gain and contrast conditions. Cells were separated into two groups: predominantly nuclear (N > C) or equal/ predominantly cytoplasmic (N ≤ C) and quantified according to [16].

### Proteomic, gene ontology (GO) and interactome analyses

#### Co-immunoprecipitation/mass spectrometry

MCF-10A cells were treated as shown on Fig. 4A. In order to maintain protein–protein interactions, cells were sonicated (two times of 7 s with 20 s of interval; 50 Hz frequency and 20% amplitude) in buffer A (1.5 mM EGTA, 1 mM EDTA, 1 mM DTT), containing protease and phosphatase inhibitors, followed by a centrifugation at 14,000 rpm and 4 °C for 15 min [19]. Supernatants were collected for subsequent immunoprecipitation assays.

Immunoprecipitation was performed by incubating 500 μg of total protein extracts with 2 μg of anti-maspin antibody or isotype matched IgG overnight at 4 °C. Samples were centrifuged (1000 rpm for 3 min at 4 °C) and antibodies were immunoprecipitated with sepharose G beads according to manufacturer's instructions. Beads were washed three times with buffer A and resuspended in 400 μL of ice-cold 100 mM Tris–HCl pH 8.5. For protein precipitation, 100 μL of cold TCA was added and proteins were allowed to precipitate overnight at 4 °C. After centrifugation (14,000 rpm for 30 min at 4 °C), pellets were washed twice with ice-cold acetone and allowed to dry at room temperature.

For protein digestion, pellets were resuspended in 30 μL of buffer B (8 M urea, 75 mM NaCl, 50 mM Tris pH 8.2), reduced with 5 mM DTT for 25 min and alkylated with 14 mM iodoacetamide for 30 min. Urea concentration in solution was reduced to 1.6 M and samples digested with trypsin (Sigma) at 1:200 ratio (enzyme:substrate) for 16 h at 37 °C. Digested peptide products were desalted using ziptips (Eppendorf) resuspended in formic acid 0.1% and injected into a C18 reversed-phase pre-column coupled to nano HPLC (NanoLC-Proxeon) using a gradient elution mode (5–35% ACN in formic acid 0.1% for 45 min followed by 35–95% ACN in formic acid 0.1% for 5 min) at 200 nL/min. The eluted peptides were analyzed online in a high-resolution mass spectrometer LTQ-Orbitrap Velos (ThermoFisher). The top 10 most intense ions were selected for CID fragmentation in data dependent analysis. Obtained data from LTQ-Orbitrap Velos were analyzed by Andromeda-MAXQUANT 1.6.1 software using the SwissProt (*Homo sapiens* downloaded on 2017—26,193 entries) database, with 20 ppm tolerance at MS and 0.5 Da at MS/MS [20, 21]. Fixed modification was cysteine carbamidomethylation and variable modifications were methionine oxidation and N-terminal acetylation, and 1% false discovery rate (FDR). At least one razor + unique peptide was considered as well as match between runs was used for peptide identification and quantification to enhance the number of reliable identifications. For quantitative values, normalized LFQ values from the output protein groups files were utilized. The final list of proteins was obtained after removing i. proteins identified at decoy and reverse database; ii. proteins identified only by modified peptides. We kept proteins present only (LFQ > 0) in at least 2 biological replicates from each group (arrest, 5 min, 15 min, 60 min, arrest-IgG control, 60 min-IgG control).

#### GO and interactome analyses

GO enrichment analysis was performed by submitting our 54 proteins identified in EGF-treated samples (gene list) to the PANTHER classification system (Fisher’s Exact test) [22]. An FDR and an arbitrary enrichment score cutoff (see inclusion criteria for each GO component analysis in Additional file 3: Table 2) were set to select the most significant biological processes, molecular functions, and cellular components. GO redundant terms were removed from GO PANTHER output list with REVIGO tool (tiny similarity option—0.4, *p-value* association and *Homo sapiens* species) [23] and highly generic terms were manually excluded to generate the final “selected GO list” (Additional file 3: Table 2). These final lists, which include each GO term and its corresponding “gene number”, “enrichment score” and “FDR” were loaded in RStudio for graphical representation according to [24]. For GO Reactome pathway analysis, we adopted a stringent cut-off (fold enrichment > 20.0 and FDR < 10^–5^) for processed list output and the remaining redundant or generic pathways were removed to generate a final “selected list” (Additional file 3: Table 2). This list was loaded into Cytoscape platform version 3.7.2 [25] for further ClueGO plug-in analysis [26]. The following parameters were set in ClueGO: functional analysis, *Homo sapiens*, visual style—significance, medium network specificity, use GO term fusion, pV < 0.0500 (Bonferroni-step down correction), organic layout. Finally, pathway networks generated were clustered with AutoAnnotate plug-in (normalization factor—0.5 and cluster cutoff—1.0) in Cytoscape. Venn diagrams [27] were built to classify proteins exclusively identified in each EGF-treated condition (Additional file 1: Fig. 3). These proteins were individually used as seed lists for Cytoscape along with corresponding peptide/unique peptides values, including maspin as interactor in each condition. Generated networks were integrated into the Mentha database (*Homo sapiens*), considering protein interactions validated by at least one experimental technique [28] (Additional file 4: table 3). Finally, the cytoHubba Plug-in was employed to identify the most important hubs in the networks by considering the MCC and Bottleneck ranking methods for the top 15 ranked proteins from the total nodes [29].

## Results

### EGF-induced maspin nuclear translocation depends on EGFR function and expression

**Fig. 2:**
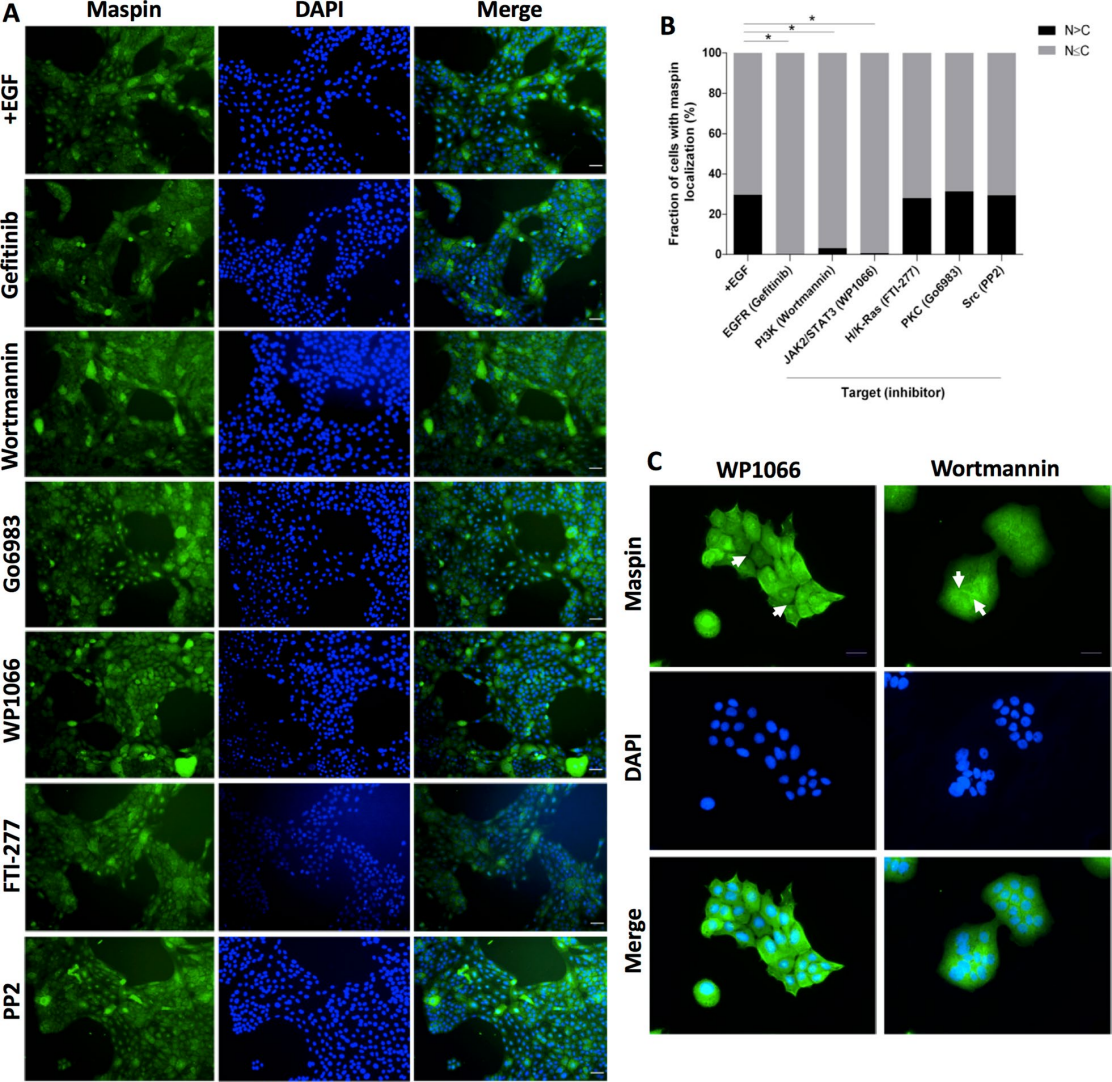
EGF-induced maspin nuclear accumulation depends on the PI3K-Akt and JAK2-STAT3 pathways **A** Starved MCF-10A cells were pretreated with inhibitors of EGFR (Gefitinib), PI3K (Wortmannin), pan-PKC (Go6983), JAK2/STAT3 (WP1066), H/K-Ras (FTI-277) and Src family kinases (PP2) (except for the upper row), for 30 min followed by 20 ng/ml EGF treatment for additional 1 h. Maspin localization was analyzed by immunofluorescence with anti-maspin antibody. Nuclei were stained with DAPI. **B** Cells were quantified based on the criteria shown next to the graph *p < 0.05 (Chi-square test). **C** The experiment described in A was repeated and images were acquired in a higher resolution. JAK2/STAT3 inhibition by WP1066 leads to a junctional discontinuity pattern, whereas PI3K inhibition by Wortmannin results in continuous maspin distribution across cell–cell junctions (arrows). Images are representative of at least three independent assays. Scale bar: 20 µm

We have reported that EGF leads to maspin phosphorylation and nuclear translocation in MCF-10A cells [16]. Immunofluorescence analysis revealed that nuclear maspin increases within 30 min of EGF treatment in MCF-10A cells, reaching its highest levels at 60 min [16]. In order to confirm this finding, serum and growth factor-starved MCF-10A cells (henceforth named simply as starved MCF-10A cells) were treated with EGF for 15, 30 and 60 min. Nuclear and cytoplasmic fractions were prepared and maspin protein levels were analyzed by immunoblot. In agreement with our previous observation, maspin nuclear accumulation followed the same kinetics (Fig. 1A, upper panel). Fractionation efficiency was confirmed by reprobing the membrane with anti-lamin A/C and anti-α-tubulin antibodies (Fig. 1A, middle and lower panels, respectively). To investigate which are the intracellular signaling pathways involved in EGF-induced maspin nuclear accumulation, we first confirmed a direct role of EGFR in this process. For this purpose, starved MCF-10A cells were treated with EGF in the presence of the EGFR inhibitor gefitinib, which inhibits EGFR tyrosine kinase by competitively blocking the ATP binding site [30]. Maspin subcellular distribution was analyzed by immunofluorescence. We found that EGFR pharmacological inhibition hampers maspin nuclear translocation (Figs. 1C and 2B). In order to confirm this finding, MCF-10A cells were transfected with two different siRNA sequences against EGFR (named here as 28i and 30i) and Stealth Select RNAi negative control. Immunoblot assay confirmed efficient EGFR knockdown (Fig. 1D). In agreement with the previous result, EGFR knockdown inhibited EGF-induced maspin nuclear accumulation (Fig. 1E). Maspin interacts with β1 integrin [2], which is functionally coupled with EGFR in different cell types [31–33]. These data prompted us to investigate if maspin interacts with EGFR in MCF-10A cells. Starved MCF-10A cells were treated with EGF for 1 h or left untreated. Whole cell extracts were immunoprecipitated with anti-maspin or anti-EGFR antibodies. The immunoprecipitated material was analyzed by immunoblot (Fig. 1B). A small but consistent amount of EGFR was immunoprecipitated with maspin irrespective of EGF treatment (Fig. 1B, upper panel), whereas no maspin could be detected in precipitated EGFR immunocomplexes (Fig. 1B, lower panel). We next wondered if maspin subcellular localization would be regulated by EGFR signaling in a different cell type. For this purpose, we took advantage of the HaCaT cells, a non-transformed human keratinocyte cell line which endogenously expresses maspin [34]. HaCaT cells were serum-starved for 24 h followed by EGF treatment for 15, 30 and 60 min. Maspin subcellular distribution was analyzed by immunofluorescence (Additional file 1: Fig. S1A). Similarly to what we have observed in MCF-10A cells [16], starved HaCaT cells exhibited a diffuse distribution of maspin in the cytoplasm and nucleus. Upon EGF treatment, maspin accumulation was first detected in 30 min and increased further at 60 min (Additional file 1: Fig. S1B). These results indicate that maspin nuclear translocation in EGF-treated cells depends on EGFR catalytic activity and expression. This process is not restricted to MCF-10A cells, as we observed similar results in HaCaT keratinocyte cells.

### PI3K-Akt and JAK2/STAT3 pathways mediate maspin nuclear accumulation

EGFR activates the Ras-Erk, PLC-PKC, JAK/Src-STAT3 and PI3K-Akt signaling pathways [35]. We used specific pharmacological inhibitors [36] of EGFR downstream pathways to examine how EGFR affects maspin subcellular localization. Starved MCF-10A cells were pretreated with different inhibitors for 30 min, followed by EGF treatment for 1 h. Maspin nuclear accumulation was determine by immunofluorescence [37]. Inhibitors of H/K-Ras, PKC and Src pathways (FTI-277, Go6983 and PP2, respectively) did not significantly interfere with EGF-induced maspin nuclear accumulation (Figs. 2A and B). On the other hand, inhibitors of JAK2/STAT3 and PI3K kinases (WP1066 and Wortmannin, respectively) inhibited this process (Fig. 2A and B). Interestingly, even though inhibition of these two pathways resulted in similar reduction of maspin accumulation in the nucleus (Fig. 2B), the pattern of maspin distribution in these conditions differs importantly (Fig. 2C). When the PI3K was inhibited, maspin distribution appears diffuse and continuous across cell–cell junctions. This pattern is often observed in untreated starved cells. In contrast, when JAK2-STAT3 was inhibited, we observed a junctional discontinuity, which becomes evident by narrow “empty spaces” among adjacent cells (Fig. 2C, arrows). Together, these results suggest that both JAK2-STAT3 and PI3K modulate maspin nuclear accumulation. In addition, maspin cytoplasmic pattern is differently affected when each of these two pathways are inhibited.

**Fig. 3:**
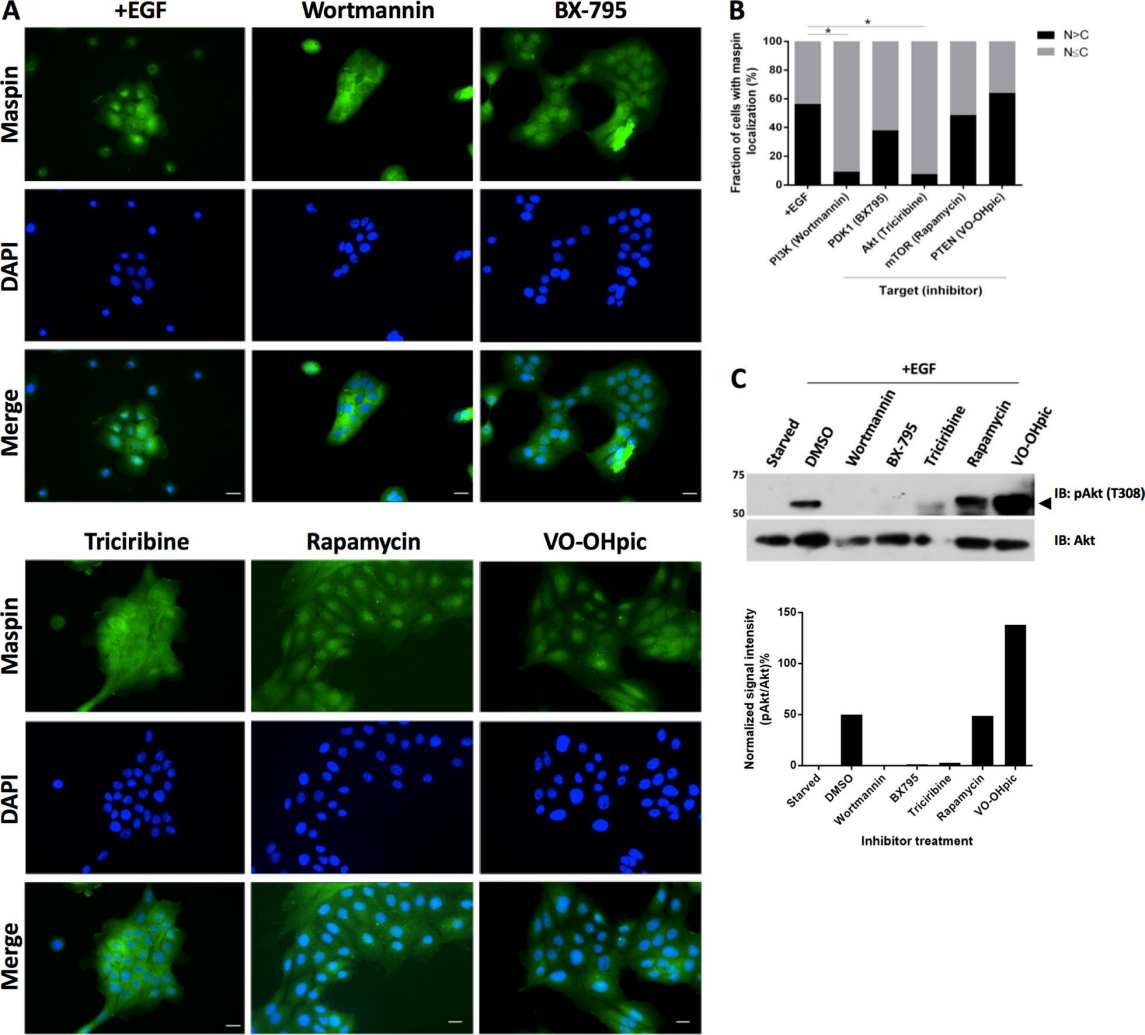
PI3K and Akt, but not PDK1, regulate maspin nuclear accumulation. **A** Starved MCF-10A cells were pretreated with inhibitors of the indicated signaling molecules (except for the three panels on the upper set, left column) for 30 min followed by EGF treatment (20 ng/mL) for additional 1 h. Maspin localization was analyzed by immunofluorescence with anti-maspin antibody. Nuclei were stained with DAPI. **B** Cells were quantified based on the criteria shown next to the graph *p < 0.05 (Chi-square test). **C** Starved MCF-10A cells were treated as described in A. Cell lysates were analyzed by immunoblot with anti-phospho-Akt (T308) antibody (upper panel, arrow). The membrane was reprobed with anti-Akt (lower panel). MW markers are indicated on the left side of the image. The pAkt and total Akt bands were quantified and pAkt/Akt ratios were plotted on a graph. Images are representative of at least three independent assays. Scale bar: 20 μm

EGFR leads to PI3K activation, resulting in recruitment of PDK1 and Akt to the plasma membrane and PDK1-mediated phosphorylation of Akt on Thr308. This process is opposed by the phosphatase PTEN. One of the key effectors of Akt is mTORC1. We used specific pharmacological inhibitors of these molecules to examine how PI3K signaling pathway regulates maspin nuclear accumulation. For this aim, starved MCF-10A cells were treated with inhibitor of PI3K (Wortmannin), PDK1 (BX-795), Akt (Triciribine), PTEN (VO-OHpic) or mTORC1 (Rapamycin) for 30 min, followed by EGF treatment for additional 1 h. Before immunofluorescence experiments, efficiency of pharmacological inhibition was confirmed by probing for phopho-Thr308 by immunoblot (Fig. 3C, upper panel). As expected, phospho-Akt (Thr308) was promptly detected upon EGF treatment, but not in starved cells. Akt phosphorylation consistently decreased upon PI3K and PDK1 inhibition. As Triciribine blocks Akt recruitment to the plasma membrane, this drug also leads to a decrease on phospho-Thr308. Consistent with an Akt effector, inhibition of mTORC1 by Rapamycin did not change Akt phosphorylation. Finally, an increase in pAkt levels was observed when PTEN was inhibited by VO-OHpic, as this phosphatase antagonizes PI3K signaling (Fig. 3C, graph). We next investigated maspin subcellular localization in the presence of individual inhibitors by immunofluorescence (Fig. 3A and B). We observed that Wortmannin and Triciribine, but not BX-795, Rapamycin or VO-OHpic, were able to consistently decrease maspin nuclear accumulation. Although PDK1 and PTEN inhibition resulted in a modest decrease and increase in nuclear maspin accumulation, respectively, they did not reach statistical significance (Fig. 3B). Altogether, these data suggest that PI3K acts via Akt modulating maspin nuclear accumulation.

### Proteomic and interactome analyses identified novel putative maspin ligands upon EGFR activation

**Fig. 4:**
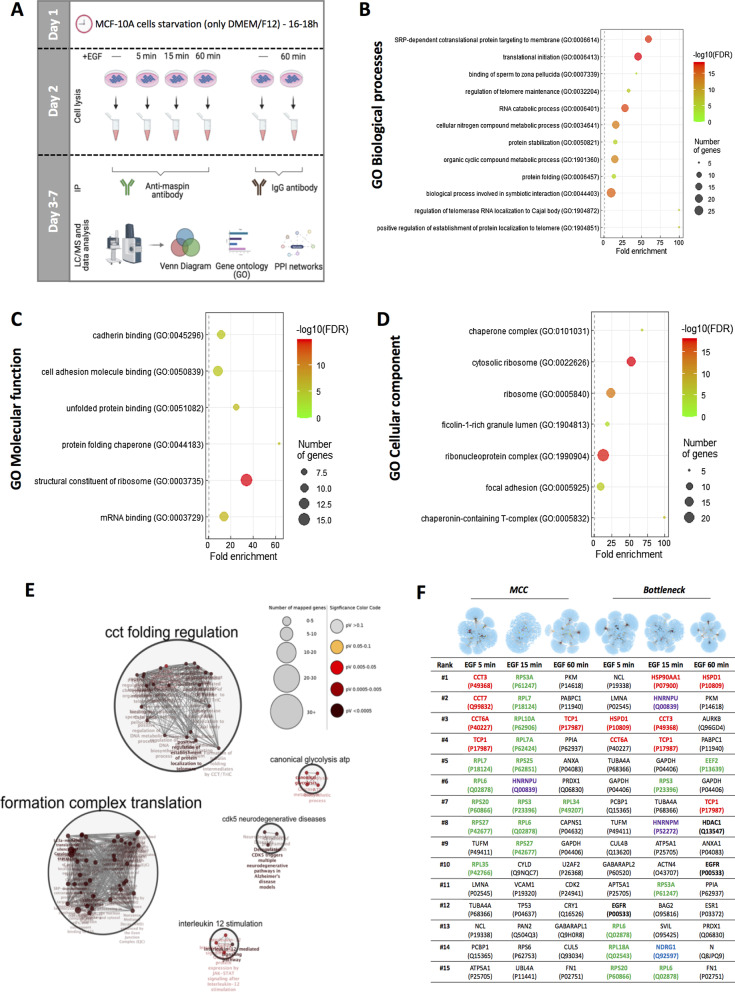
Screening for maspin ligands upon EGF treatment in MCF-10A cells. **A** Experimental strategy used to identify maspin ligands by IP/MS. Starved MCF-10A were left untreated or treated with EGF for indicated periods of time. After IP/MS analysis, proteins exclusively found in EGF-treated samples were classified using Venn diagram, GO annotation and protein–protein interaction (PPI) networks. **B–D** GO enrichment analysis displaying associated biological process, molecular function and cellular component (curated by PANTHER classification system). Fisher’s exact-test was performed with an FDR < 0.05. **E** Reactome pathway analysis showing the most significant pathways for the identified maspin ligands. Pathways were grouped into functional clusters with ClueGO plug-in. **F** Interactome analysis with maspin ligands performed by Cytoscape. Generated networks for 5, 15 and 60 min were integrated into the Mentha database (*Homo sapiens*), which integrates protein interactions already validated by at least one experimental technique (5 min—2619 nodes; 15 min—2899 nodes; 60 min—1620 nodes). Maximal Clique Centrality (MCC) and Bottleneck topological analyses performed by Cytohubba plug-in showing the top 15 central nodes in corresponding networks. The ligands associated with folding and unfolding (red), translation (green), transcription (purple), EGFR/cell–cell adhesion (blue) and already known maspin ligands (black-bold) are highlighted

Our objective was the identification of maspin ligands involved in maspin nuclear translocation or in its nuclear-associated functions using proteomic and interactome analyses in the MCF-10A cell model. For this purpose, starved MCF-10A cells were left untreated or treated with EGF for 5, 15 and 60 min (Fig. 4A). Cell lysates were incubated with anti-maspin antibody to precipitate maspin immunocomplexes. An isotype-matched IgG antibody was used as a negative control. Venn diagram was built to classify maspin ligands exclusively found in EGF-treated samples (Additional file 1: Fig. S3). Maspin was consistently immunoprecipitated from starved and EGF-treated samples (see Additional file 2: Table S1 for a list of all identified ligands).

The categorization of the 54 identified maspin ligands upon EGF treatment (Additional file 2: Table S1) was made by PANTHER classification system, processed by REVIGO tool, as depicted in Fig. 4B–D (Additional file 3: Table S2). The GO enrichment analysis revealed maspin interactors in biological processes such as SRP-dependent cotranslational protein targeting to membrane, translation initiation, RNA catabolic process and regulation of telomerase RNA localization to Cajal bodies. Accordingly, specific GO molecular functions described the maspin-associated ligands to structural constituents of ribosomes, RNA-binding, unfolded protein binding and cell adhesion binding, including those mediated by cadherins. Interestingly, GO cellular component profile indicated a predominance of ribonucleoprotein and ribosomal complexes, chaperone complex and focal adhesion. To gain further insight into which pathways maspin ligands are involved, we employed Reactome analysis (Additional file 3: Table S2) with a stringent cut-off (pV < 0.05) in ClueGO plug-in and grouped the main pathways found using AutoAnnotate in Cytoscape (Fig. 4E). Most identified pathways relate to chaperonin folding (CCT folding regulation cluster), RNA metabolism and protein translation (formation complex translation cluster) and glycolysis (canonical glycolysis ATP cluster). Indeed, among all identified ligands, only TCP-1 (member of chaperonin-containing T-complex), RPL18A (60S ribosomal protein L18a) and GAPDH (glyceraldehyde-3-phosphate dehydrogenase) were found in all EGF-treated samples according to Venn diagram (Additional file 1: Fig. S3).

We then generated protein–protein interaction networks with all ligands identified in each EGF-treated sample using Cytoscape platform. The seed networks were merged to the Mentha database (Additional file 4: Table S3), which integrates protein interactions already validated by at least one experimental technique. Topological analyses were performed with the Cytohubba plug-in and identification of the top 15 central hubs were carried out using at least two different methods based on the shortest paths, the Maximal Clique Centrality (MCC) and the Bottleneck (Fig. 4F).

The obtained results for MCC analysis showed a predominance of TRiC complex subunits at 5 min (#1 CCT3, #2 CCT7, #3 CCT6A and #4 TCP-1) and 60 min (#3 TCP1) following EGF treatment (Fig. 4F). In addition, a set of ribosomal proteins were consistently ranked at 5 and 15 min time points. Finally, hnRNPU (#6) was found at 15 min, reinforcing its relevance already seen in enriched members of hnRNP family in the proteomic results at this time point.

In order to gain further insight on other relevant maspin potential ligands within the generated networks, we complemented our MCC analysis using the Bottleneck method. MCC analysis is a local-based method whereby only the relationship between the nodes and its direct neighbors is considered. On the other hand, Bottleneck is a global-based method where the relation between the node and the entire network is evaluated [29]. Indeed, Bottleneck analysis highlighted members of the TRiC complex also at 5 min (#4 CCT6) and at 15 min (#3 CCT3; #4 TCP-1) (Fig. 4F). In addition, riboproteins were consistently ranked in 5 (#6 RPL6) and 15 min (#8 RPL6) samples and once again ribonucleoproteins (#2 hnRNPU; #8 hnRNPM) were detected at 15 min. More interestingly, proteins simultaneously involved in cell adhesion and EGFR signaling were detected, such as NDRG1 (#14) at 15 min. Although EGFR was not detected in any of our proteomic conditions, the Bottleneck method ranked it in 5 min (#12) and 60 min (#10) samples. The reason why EGFR was not captured bound to maspin is likely due to the fact that our cell extracts for proteomic analysis were obtained without any detergent, which hampers the solubilization of membrane-bound receptors such as EGFR while preserves protein–protein interactions. Despite that, EGFR/maspin interaction seems to be relatively stable, as reciprocal co-immunoprecipitation was carried out in a much more stringent lysis buffer (Fig. 1B). Finally, HDAC1 and HSP90, which are known maspin ligands [38, 39], were ranked at 60 min (#8) and 15 min (#1), validating our experimental approach.

### A role for cell–cell contact in maspin nuclear accumulation

**Fig. 5:**
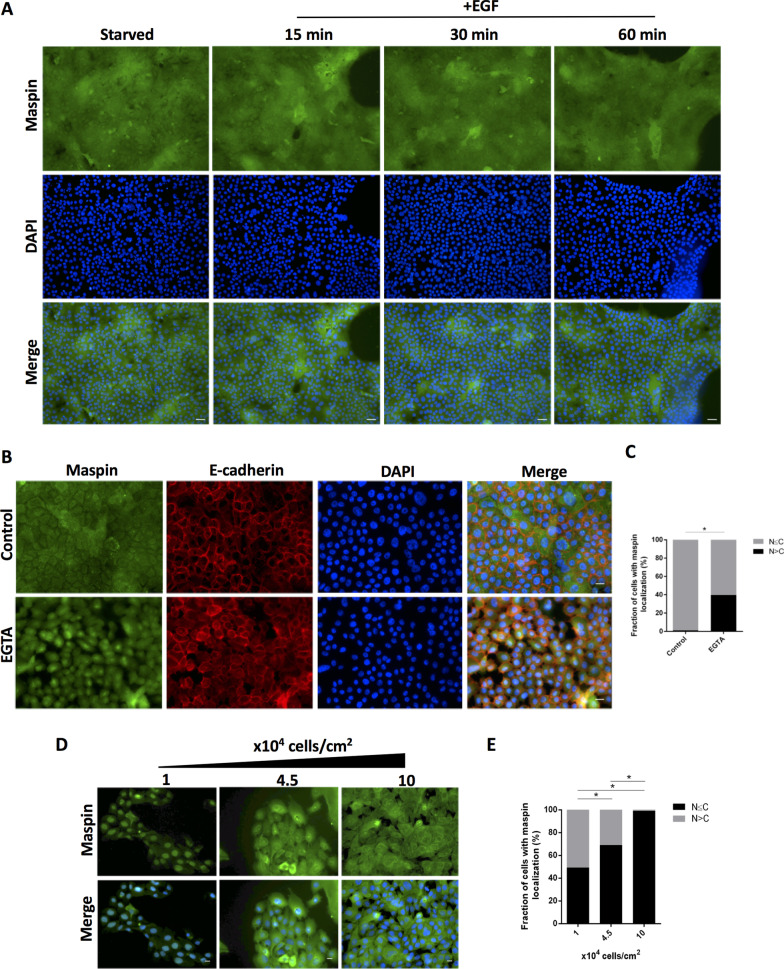
Cell–cell contact regulates maspin nuclear translocation in MCF-10A cells. **A** MCF-10A cells were plated at high confluency. The next day, cells were starved for 24 h followed by 20 ng/ml EGF treatment for the indicated intervals. Maspin distribution was analyzed by immunofluorescence **B** MCF-10A cells were treated with 4 mM of EGTA for 30 min. Maspin and E-cadherin patterns were examined by immunofluorescence. **C** Cells were quantified based on the criteria shown next to the graph *p < 0.05 (Chi-square test). **D** MCF-10A cells were plated at the indicated cell densities and maspin localization was analyzed by immunofluorescence. **E** Cells were quantified based on the criteria shown next to the graph, *p < 0.05 (Chi-square test). Images are representative of at least three independent assays. Scale bar: 20 μm

We have observed for some time that regardless of cell culture condition or pharmacological treatment, maspin subcellular localization is heterogeneous throughout the dish surface when culture displays low to mid confluency (Fig. 2A, left column). This variation appeared to be related to the fact that MCF-10A cells tend to form clusters [40]. After reaching confluency, however, maspin staining acquires a cloudy homogeneous pattern across the cytoplasm and nucleus (Fig. 5A, upper row). Interestingly, when confluent MCF-10A cells were treated with EGF, maspin distribution remains unchanged even after 1 h of EGF treatment (Fig. 5A). Since cell–cell adhesion in epithelial cells is mediated by E-cadherins, and these are calcium-dependent cell adhesion molecules, we examined whether calcium removal would affect maspin subcellular localization. To this end, MCF-10A cells were treated with 4 mM EGTA, a calcium chelating agent, for 30 min at 37 °C. E-cadherin and maspin were analyzed by immunofluorescence. We observed that calcium removal redirects maspin to the nucleus (Figs. 5B and C). To gain further support for this hypothesis, MCF-10A cells were plated in complete medium at different densities and maspin subcellular localization was determined by immunofluorescence. In agreement with previous observations, in sparse cells maspin is predominantly nuclear, but it becomes cytoplasmic as cell density increases (Figs. 5D and E). To make sure this is not a process limited to MCF-10A cells, we repeated the experiment using HaCaT keratinocytes (Additional file 1: Fig. S2A and B). Similarly, maspin is mostly found in the nuclei of sparse cells, becoming cytoplasmic as cell density increases. Together, these results demonstrated that maspin nuclear localization is regulated by calcium-dependent cell–cell contact. In addition, cell–cell contact appears to be dominant over EGF stimulus, at least in this experimental condition. Finally, this process is not restricted to MCF-10A cells, as it was also observed in HaCaT keratinocytes.

## Discussion

We have previously reported that EGF leads to maspin phosphorylation and nuclear accumulation in the MCF-10A model system. Here we began to unravel how maspin subcellular localization is regulated. We observed that EGFR physically interacts with maspin and EGFR expression and kinase activity are essential for maspin nuclear accumulation. The kinetics of maspin translocation, which was first observed by immunofluorescence [16] was here confirmed by subcellular fractionation. Using specific pharmacological inhibitors, we found that two important EGFR downstream pathways—PI3K-Akt and JAK2-STAT3, are essential in this process. In addition, we provide evidence that signals emanating from cell–cell contact act in concert with EGFR signaling regulating maspin nuclear-cytoplasmic distribution. As EGFR signaling acts on a plethora of cellular processes, we conducted proteomic and interactome analyses in EGF-treated MCF-10A cells to investigate in which of these processes maspin is involved. We uncovered a novel set of putative maspin ligands which suggest that maspin is involved in post-transcriptional and translation regulation, protein folding and cell–cell adhesion.

### Signals controlling maspin subcellular localization

Our data show that while EGF treatment results in maspin nuclear accumulation in low-mid confluent culture (Fig. 1C) [16], it has no effect in confluent culture, as if cells had become resistant to EGF (Fig. 5A). This resistance appears to be opposed by calcium removal (Fig. 5B and C). The role of cell–cell contact in maspin cellular distribution was most evident when cells were plated in increasing cell densities (Fig. 5D). Together, these results suggest that signals derived from calcium-dependent cell–cell contact are dominant over those derived from EGFR when it comes to maspin subcellular distribution. We made a similar observation in HaCaT keratinocytes (Additional file 1: Figs. 1 and 2), suggesting that this process may be a common feature of epithelial cells. EGFR and E-cadherin complex keep a reciprocal and dynamic interrelationship which has been extensively investigated in different model systems [41]. Interestingly, they share important downstream targets: E-cadherin engagement leads to Rac and PI3K-Akt activation [42, 43], and both Rac and PI3K leads to STAT3 activation [44, 45]. EGFR directly activates PI3K, which can activate Rac via GEF-Racs [46]. Thus, the inhibition of EGFR signal by cell–cell contacts, when it comes to maspin distribution, likely involves differences in signal intensity and spatiotemporal activation. Actin cytoskeleton architecture, which is regulated by Rac [47], is major target of EGFR and E-cadherin signaling. Different studies reported that maspin leads to Rac activation and actin cytoskeleton remodeling [48, 49]. In MCF-10A cells, maspin is associated with the detergent-insoluble cortical cytoskeleton [2] and one study found that maspin can directly interact with actin [50]. These data suggest that actin cytoskeleton may be an important element underlying maspin subcellular distribution downstream of E-cadherin and EGFR crosstalk. Our previous observation [16] and the kinetics of maspin nuclear translocation in EGF-treated cells (Fig. 1A) show that while a fraction of maspin translocates to the nucleus, most of it remains in the cytoplasm. We observed that maspin cytoplasmic pattern differs between PI3K and JAK2-STAT3-inhibited cells—maspin looks cloudy and continuous across cell–cell junctions when PI3K is inhibited. This continuity is somehow disrupted when JAK2-STAT3 is inhibited, which is evidenced by gaps among cells (Fig. 2C, arrows). These results suggest that although inhibition of PI3K and JAK2-STAT3 resulted in comparable inhibition of maspin nuclear accumulation (Fig. 2A and B), these pathways interfere differently in the organization of cytoplasmic maspin. We presently do not understand how maspin cytoplasmic pattern is regulated and the biological processes related to it. Since actin cytoskeleton is an essential target of the pathways mentioned above, their inhibition likely results in alteration in epithelial cell shape. If cells tend to flatten, gaps will appear among them; if they become more columnar, the lateral membranes of adjacent cells will share a larger surface, and gap will not be visible. It would be interesting to examine if cytoplasmic maspin is reorganized in response to cell shape.

Our immunofluorescence experiments show that maspin intracellular distribution is heterogeneous among different cells. The heterogeneity does not appear to depend on culture conditions or pharmacological inhibition (Figs. 1C and 2A, left columns). It has been reported that Akt activation in MCF-10A depends on total PI3K protein levels in the cell, so that only subpopulations with higher PI3K expression efficiently activate Akt when cells are treated with EGF. Interestingly, PI3K protein turnover is regulated by cell density—increasing cell–cell contacts drives these cells to the so-called PI3K-low state, and therefore Akt activation is low. Conversely, in low cell density Akt is more prone to activation due to high levels of PI3K (PI3K- high state) [51]. These data suggest that cell–cell contact may modulate maspin distribution in the cell by regulating PI3K protein levels, which in its turn define the level of Akt activation. It has long been believed that upon PI3K activation, a dual regulatory mechanism is essential for full activation of Akt. This mechanism depends on Akt phosphorylation at T308 and S473 residues by PDK1 and mTOR kinases, respectively [52]. In our immunofluorescence experiments, PI3K and Akt inhibition, but not PDK1, hampered maspin nuclear accumulation (Figs. 3A and B). The same observation has been done in insulin-dependent GLUT4 translocation to the cell surface in 3T3-L1 adipocytes [53]. We speculate that PI3K and Akt may act independently in this process [54] since Akt may be activated by different mechanisms [55].

### Maspin subcellular localization in cancer

The association between maspin tumor suppressor activity and its subcellular localization is complex and not completely understood. A correlation between nuclear or pan-cellular maspin and a good prognostic has been reported in breast [56], ovarian [57] and laryngeal tumors [58, 59] but not in pancreas [9, 60], skin [61] and colorectal cancer [62]. Different methodologies and/or scoring systems, besides the remarkable differences among tumors are likely responsible for these discrepant reports. In addition, these divergences underscore the value of understanding how maspin subcellular localization is regulated in a non-tumoral model system. Numerous studies which used tumor cells lines to examine maspin biological activities in general support a role of maspin in tumor suppression [63]. Clinical studies, however brought divergences to the field, suggesting that tissue context and microenvironment may influence maspin tumor suppression activity. Our observation that maspin subcellular localization is under cell–cell contact control further supports this hypothesis. Two studies specifically addressed the effect of nuclear maspin in tumor cell culture. One of them observed an inhibition of human breast tumor growth and metastasis in xenograft models [10] and the other observed reduced cell proliferation [5]. These results suggest that nucleus-directed maspin may be used as an anticancer agent in gene therapy. We showed here that maspin subcellular localization is controlled by EGFR, JAK-STAT3 and PI3K-Akt pathways, which are often dysregulated in tumorigenesis and cancer progression [64–66]. These pathways are important targets for therapy, but they are also responsible for drug resistance [67, 68]. In this context, maspin subcellular localization emerges as a candidate biomarker to predict resistance.

### Identification of new maspin ligands associated with EGFR activation

Because EGFR signaling regulates multiple cellular processes, we set out to investigate in which of these processes maspin would be involved (Fig. 4A). Unexpectedly, many of the new maspin partners are involved in processes which have not been previously associated with maspin. Notably, we found structural constituents of ribosomes, RNA-binding proteins, focal adhesion molecules and chaperonins (Figs. 4B, C and D). The T-complex protein-1 Ring Complex (TRiC complex) comprises eight different subunits [69], five of which interact with maspin. The TRiC is in charge of folding approximately 10% of cellular proteins, among them are actin and tubulin [70]. TRiC prefers proteins with domains that have trouble folding, like maspin's β-strand-enriched regions [71]. We have recently described a nuclear localization signal (NLS) in maspin polypeptide sequence, which is, however, cryptic [72]. Post-translational modifications such as phosphorylation could assist in exposing maspin NLS. An interesting testable hypothesis would be that upon EGFR activation, maspin interacts with the TRiC complex, which would lead to its NLS exposure and subsequent nuclear translocation. Accordingly, TRiC is responsible for the correct folding and nuclear translocation of the telomerase cofactor TCAB1 [73]. Consistent with our results shown here, we identified maspin ligands which were grouped as cell–cell adhesion mediated by cadherins in GO analysis (Fig. 4C). Another interesting new maspin ligand is NDRG1, a protein which shares similarity to maspin in stimulating apoptosis [74], reducing angiogenesis [75], cell proliferation [76] and migration [77]. In addition, NDRG1 regulates cell–cell adhesion and cell growth and proliferation mediated by EGFR/Akt/PTEN pathway [78]. Finally, we identified five hnRNPs (D, U, H, M and E) which are part of the spliceosome, and several ribosomal proteins (Fig. 4C). Interestingly, it has been shown that EGF is responsible for reprogramming splicing in the nucleus through Akt and JAK/STAT in coordination with HSP70/HSP90 [79]. These results open new perspectives in understanding the molecular mechanism underlying maspin multiple biological function, providing unique opportunities for therapeutic intervention.

## Conclusions

We identified three different molecular nodes—PI3K-Akt, JAK2-STAT3 and cell–cell contact regulating maspin nuclear translocation. These multiple levels of regulation underscore the importance of maspin subcellular localization on its diverse biological activities. In addition, we identified endogenous maspin ligands which brought new insight into how maspin control these activities. The molecular and functional implications of some of these maspin interactors are currently under investigation by our group. We thus hope this leads to advances in the use of maspin for therapeutic or prognostic purposes in order to rationally address breast cancer disease.

## Supplementary Information

**Additional file 1. Supplemental figures 1** (EGF treatment leads to maspin nuclear accumulation in HaCaT cells), **2** (Cell-cell contact regulates maspin nuclear translocation in HaCaT cells) and **3** (Venn diagram for EGF-treated samples).

**Additional file 2**.** Table S1**: All maspin ligands identified by co-IP/MS

**Additional file 3**. ** Table S2**: Gene ontology and Reactome analyses for maspin ligands identified in EGF-treated samples

**Additional file 4**.** Table S3**: Protein–protein interaction network analysis for maspin ligands identified in EGF-treated samples

## Data Availability

The datasets supporting the conclusions of this article are included within the article and its additional files. The mass spectrometry proteomics data have been deposited to the ProteomeXchange Consortium via the PRIDE partner repository with the dataset identifier PXD023224.

## Abbreviations

EGF: Epidermal growth factor
EGFR: Epidermal growth factor receptor
JAK2: Janus kinase 2
STAT3: Signal transducer and activator of transcription
PI3K: Phosphoinositide 3-kinases
mTORC: Mammalian target of rapamycin complex
PDK1: Phosphoinositide-dependent kinase-1
PTEN: Phosphatidylinositol 3,4,5-trisphosphate 3-phosphatase
PKC: Protein kinase C
EGTA: Ethylene glycol tetraacetic acid
ERK: Extracellular-signal-regulated kinase
MAPK: Mitogen activated protein kinase
Rac: Ras-related C3 botulinum toxin substrate 1
hnRNPs: Heterogeneous nuclear ribonucleoproteins
HDAC1: Histone deacetylase 1
HSPs: Heat shock proteins
TRiC: T-complex protein-1 Ring Complex
NDRG1: N-myc downstream regulated gene 1
MCC: Maximal clique centrality
