# Supplementary material for: PI3K-AKT, JAK2-STAT3 pathways and cell–cell contact regulate maspin subcellular localization
Source: Cell Commun Signal. 2021 Aug 14;19:86. doi: 10.1186/s12964-021-00758-3 (PMC8364028; doi:10.1186/s12964-021-00758-3)
Supplement: Supplementary file 2 — Additional file 1. Supplemental figures 1 (EGF treatment leads to maspin nuclear accumulation in HaCaT cells), 2 (Cell-cell contact regulates maspin nuclear translocation in HaCaT cells) and 3 (Venn diagram for EGF-treated samples). [file 12964_2021_758_MOESM1_ESM.docx]

Supporting information for:

**PI3K-AKT, JAK-STAT3 pathways and cell-cell contact regulate maspin nuclear localization**

Longhi MT^1†^, Silva LE^1†^, Pereira M^1^, Magalhães M^1^, Reina J^1^, Vitorino FNL^3^, Gumbiner B^2^, da Cunha JPC^3^ and Cella N^1*^

Supplemental figure 1:


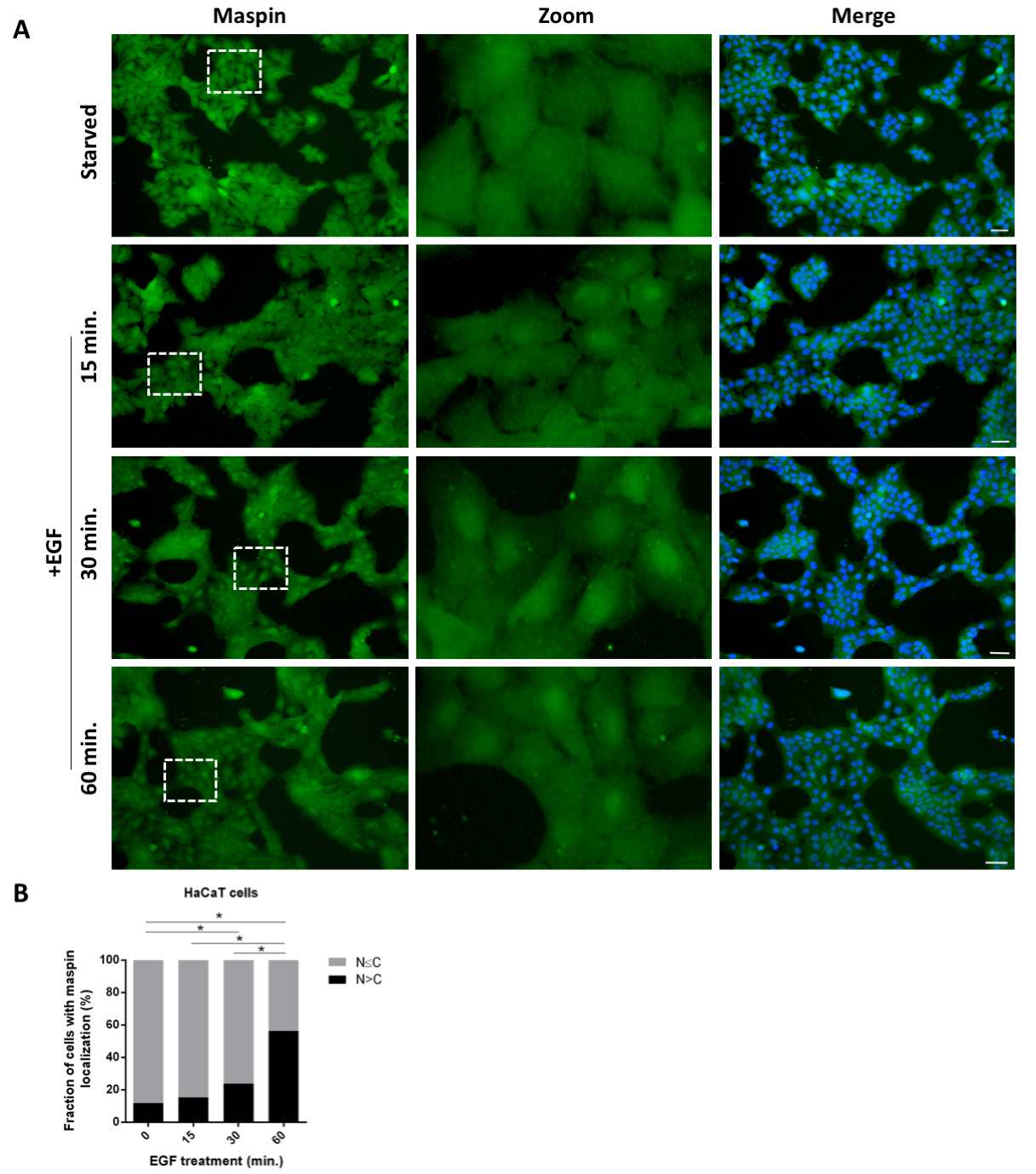


**Figure 1 – EGF treatment leads to maspin nuclear accumulation in HaCaT cells. A.** HaCaT cells (non-transformed immortalized human keratinocytes) were serum starved (24 hrs) and treated with 20 ng/mL of EGF for the indicated periods of time. Cells were fixed and processed for immunofluorescence with anti-maspin antibody. Nuclei were stained with DAPI. Scale bar: 20 μm; **B.** Cells were quantified based on the criteria shown next to the graph, *p<0.05 (Chi-square test).

Supplemental figure 2:


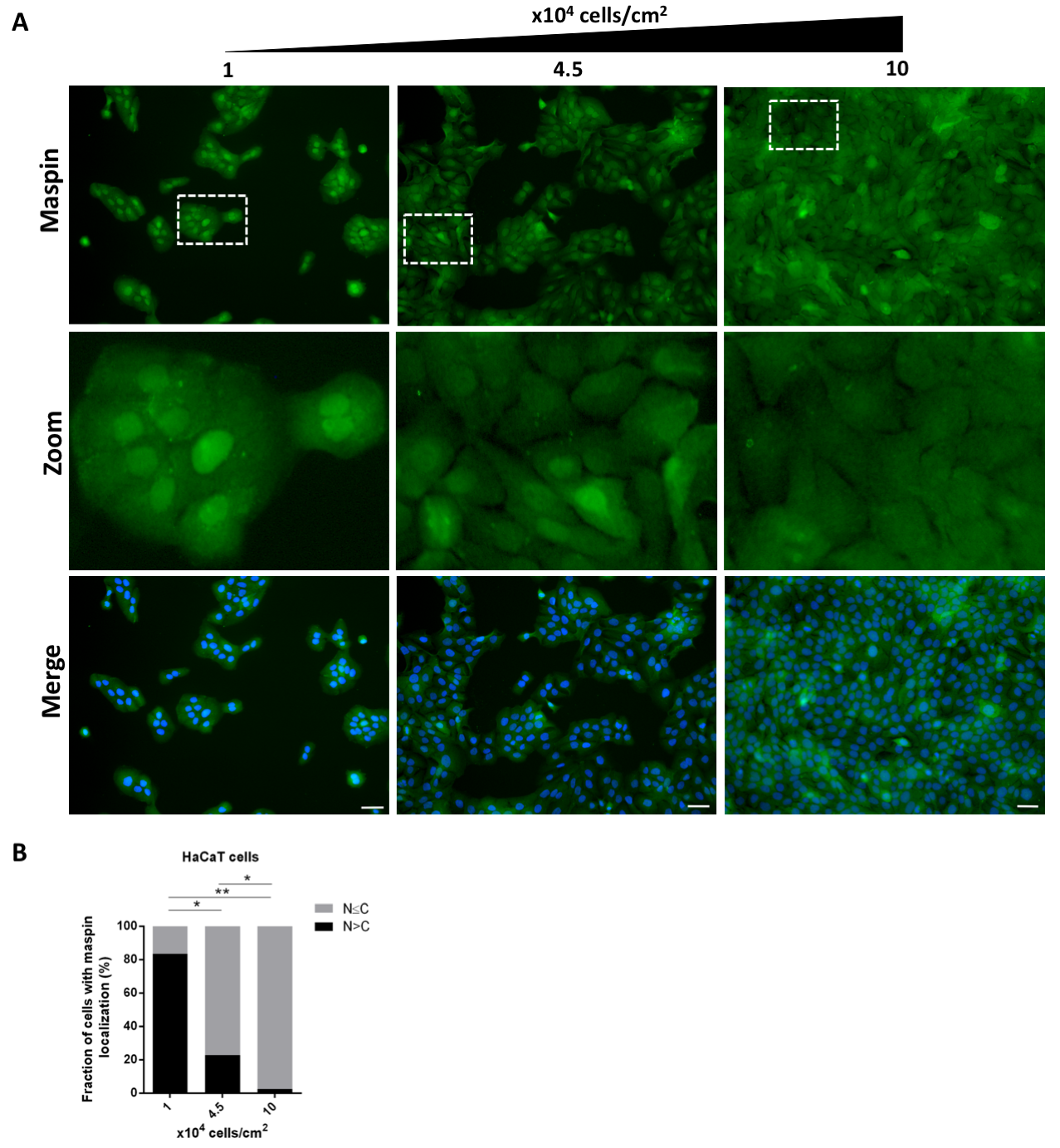


**Figure 2 – Cell-cell contact regulates maspin nuclear translocation in HaCaT cells. A.** HaCaT cells were plated at the indicated cell densities in complete medium. After 48 h, maspin localization was analyzed by immunofluorescence with anti-maspin antibody. Nuclei were stained with DAPI. Scale bar: 20 μM. **B.** Cells were quantified based on the criteria shown next to the graph, *p<0.05 (Chi-square test).

Supplemental figure 3:


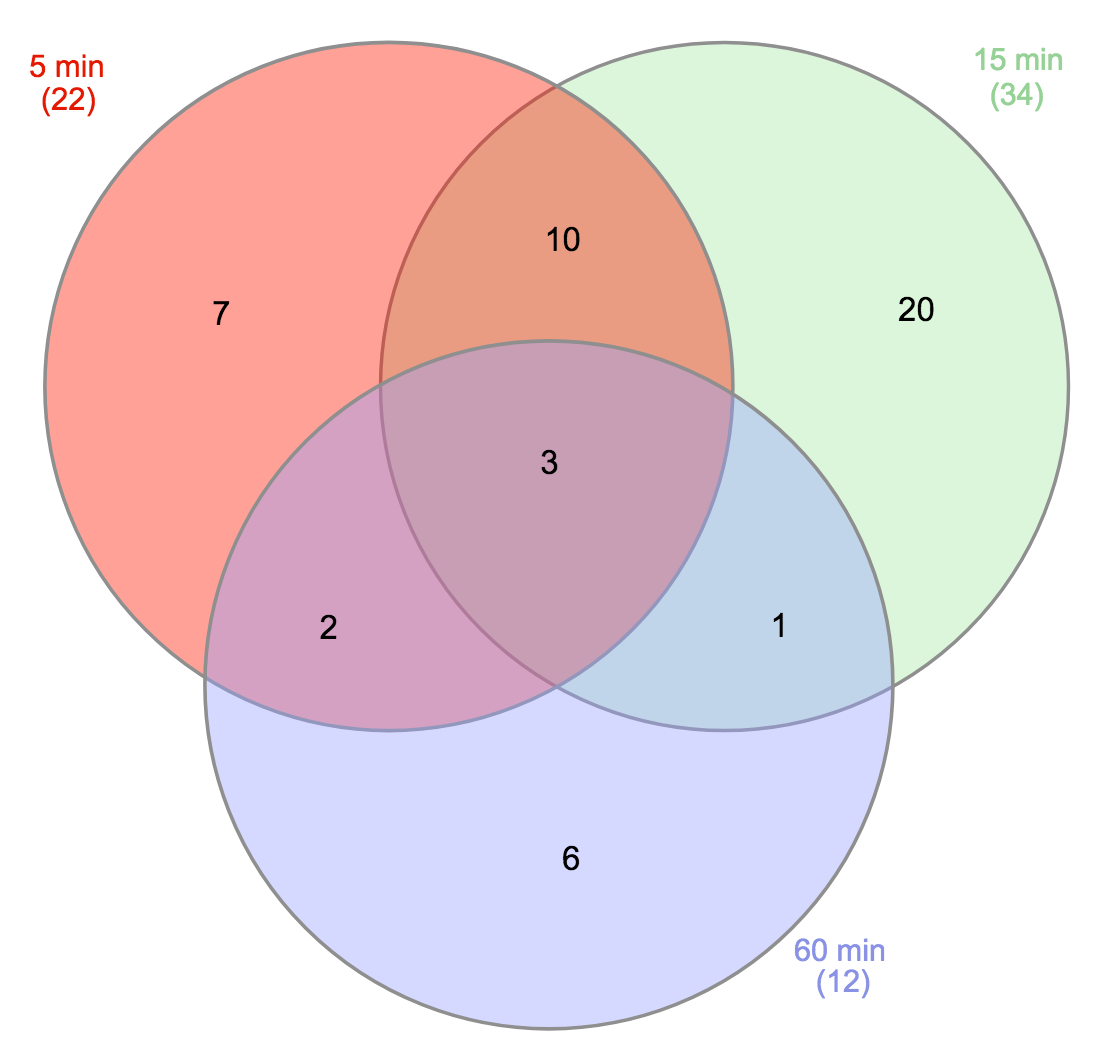


GAPDH

TCP-1

RPL18A

**Figure 3 – Venn diagram for EGF-treated samples.** Proteins exclusively found in EGF-treated samples were classified by using InteractiVenn. The black arrow indicates shared hits in all time points analyzed.
